# The Low-Cycle Fatigue Behavior, Microstructure Evolution, and Life Prediction of SS304: Influence of Temperature

**DOI:** 10.3390/ma16186326

**Published:** 2023-09-21

**Authors:** Ting Mei, Quanyi Wang, Meng Liu, Yunqing Jiang, Tongfei Zou, Yifan Cai

**Affiliations:** 1 AVIC Guizhou Honglin Aerodynamic Control Technology Co., Ltd., Guiyang 550000, China; meiting0725@sina.com; 2 Failure Mechanics and Engineering Disaster Prevention and Mitigation, Key Laboratory of Sichuan Province, College of Architecture and Environment, Sichuan University, Chengdu 610065, China; wangquanyi@stu.scu.edu.cn (Q.W.); patricial97@163.com (M.L.); jiangyq2333@outlook.com (Y.J.); ephrysz@outlook.com (T.Z.); 3 Key Laboratory of Deep Underground Science and Engineering, Ministry of Education, Sichuan University, Chengdu 610065, China

**Keywords:** SS304, tensile behavior, low-cycle fatigue, temperature, damage mechanism, life prediction

## Abstract

To study the fatigue failure and microstructure evolution behavior of SS304, low-cycle fatigue tests are conducted at room temperature (RT), 300 °C, and 650 °C. The results indicate that, because of the influence of the dislocation walls, carbon-containing precipitates, and deformation twins, the cyclic hardening behavior is presented at RT. However, different from the cyclic hardening behavior at RT, the cyclic softening behavior of SS304 can be observed due to the dynamic recovery and recrystallization containing dislocation rearrangement and annihilation at 300 °C and 650 °C. In addition, two fatigue crack initiation modes are observed. At RT, the single fatigue crack initiation mode is observed. At high temperatures, multiple crack initiation modes are presented, resulting from the degradation of material properties. Furthermore, a new fatigue life prediction model considering the temperature is conducted as a reference for industrial applications.

## 1. Introduction

One of the widely used types of chromium-nickel stainless steel, 304 austenitic stainless steel (SS304 abbreviated), has outstanding corrosion resistance, heat resistance, low-temperature strength, and mechanical properties, as well as promising machinability and weldability [1,2,3,4], and it is the primary material consumed for nuclear power used to manufacture in-stack components such as coaming bolts, voltage regulator vessels, pre-heating tubes [5,6], etc.

In recent years, research on SS304 and its derivatives under different loading methods and experimental temperatures has been carried out extensively from academia to the manufacturing industry. Meanwhile, the results indicate that the cyclic response behavior of SS304 is related to the experimental conditions and material processing technology [7,8,9,10,11] during LCF. Generally, the hardening material (quenched, cold-worked) softens cyclically, and the softening material (annealed) hardens cyclically [12]. Meanwhile, the previous studies indicate that the cyclic hardening or softening behavior depended not only on the material but also on the magnitude of the applied loading (stress/strain amplitude) and the loading path (pre-stressing/pre-cycling) [13,14,15]. Moreover, Xu, Nie, Fan, Tao, and Ding [16] observed in the LCF test on BLY160 that work-hardening is an essential hysteresis property concerning the energy dissipation of the steel. However, as plastic strain accumulates, a “flatting effect” occurs, i.e., cyclic softening in both stiffness and stress. Zhou, Sun, Kanouté, and Retraint [17] compare the cyclic hardening/softening behavior of two types of 316 L steel in uniaxial LCF at RT and establish a functional relationship among the kinematic hardening coefficient φ, the accumulated plastic strain, and the memory surface based on different strain amplitudes. Wang, Chang, Shi, Yuan, Yang, and Liao [18] investigate the mechanical properties of S31608 under cyclic loading with amplitudes ranging from ±0.4% to ±1% using eight loading schemes and calibrate the cyclic hardening model through simulation based on test data. Beyond that, many impact factors also result in the fatigue failure of SS304, such as the different production processes, a wide range of working temperatures, complex operating environments, and so on. Since high temperature can cause degradation of the microstructure and impair its mechanical properties, Xie, Jiang, Chen, Zhang, and Tu [19] indicate that the cyclic softening behavior at RT and high temperatures is driven by dynamic recovery and fatigue damage, respectively. Wei, Feng, Han, Zhang, and Zhang [20] demonstrate that the dislocation evolution of HS80H steel at 350 °C is influenced by dynamic strain aging (DSA), where dislocation slips are blocked to form dislocation cells and concert slatted substructures into smaller cellular substructures. In addition, since fatigue damage (FD) is a localized process of permanent structural change due to the initiation of cracks and continued expansion triggered by cyclic loading, fatigue life is closely linked to macro-mechanical properties. Therefore, energy-based fatigue models [21,22,23,24,25,26] for LCF life prediction are critical for structural design and reliability assessment of components. Fatigue damage localization amplifies small differences in microstructure, making the experimental fatigue life more dispersed, which requires a more stringent life prediction model. In summary, it is necessary to research the fatigue characteristics, microstructural evolution behavior, and life prediction of SS304 from RT, 300 °C to 650 °C.

As an important engineering material, 304 austenitic stainless steel is widely used in coaming bolts, voltage regulator vessels, and pre-heating tubes [5,6]. During the operation, since the mechanical components are designed for normal operating loads and superimposed cyclic loads, the cyclic loading in the plastic range results in cyclic plastic deformation of SS304. It can be described as symmetrical strain cycles caused by the temperature fluctuations in the start-up and shut-down of the mechanical components, leading to premature low-cycle fatigue failure of the workpiece [27]. Considering the loading conditions, it is critical to evaluate the fatigue behavior of SS304. Thus, in this study, to understand the cyclic response behavior, fatigue failure behavior, and microstructural evolution of SS304, systematic analyses, including uniaxial tensile tests and low-cycle fatigue tests at RT, 300 °C, and 650 °C, are conducted. To begin with, the hardening/softening behavior of SS304 was investigated by uniaxial tensile tests with a strain rate of 2.5 × 10^−3^ s^−1^ and LCF tests with various strain amplitudes, such as 0.5%, 0.6%, 0.8%, and 1.0% at different temperatures and loading patterns. Then, the experimental data were analyzed to develop a modified damage energy model based on the Manson–Coffin relationship. Finally, the fracture morphology and micro-mechanics were represented through scanning electron microscopy (SEM) and transmission electron microscopy (TEM), revealing the impact of temperature on the fatigue micro-zone mechanism.

## 2. Material and Experimental Procedure

**Figure 1:**
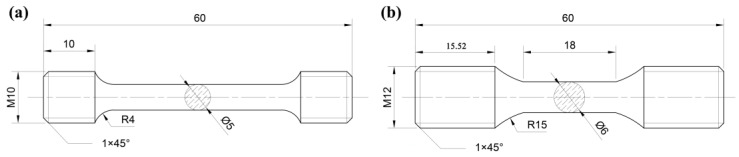
The geometry of the (**a**) tensile specimen and (**b**) low-cycle fatigue specimen (Unit: mm).

In the current work, the chemical compositions of SS304 are listed in Table 1. Tensile and LCF tests were carried out according to the standard ASTM E8M-04 [28] and ASTM E606-2012 standard [29] with cylindrical specimens designed for diameters of 5 mm and 6 mm, respectively. The detailed geometry is shown in Figure 1. To minimize the influence of processing, all specimens were mechanically polished. The surface roughness of polished specimens was about 0.4 μm, and the error was within 0.02 mm in diameter.

The tensile and LCF were performed on a high-temperature electronic universal testing machine (Shimadzu Corporation, Kyoto City, Japan) and a 50 kN SHIMADZU fatigue machine (Shimadzu Corporation, Kyoto City, Japan) at RT, 300 °C, and 650 °C, respectively. A clamped axial extensometer was provided to control and monitor the current strain. The electric resistance furnace was operated to simulate a high-temperature environment, where three K-type thermocouples were installed to check temperature fluctuations to ensure uniform heating everywhere. To guarantee a steady state at the required temperature before tests, the specimen was held for 30 min after reaching the target temperature. The strain rate of 2.5 × 10^−3^ was chosen at uniaxial tensile at three temperature levels. The triangular cyclic waves were controlled at the strain ratio R = −1 to test at strain amplitudes (∆ε/2) of 0.5%, 0.6%, 0.8%, and 1.0%. Considering that the strain rate at each strain amplitude of low-cycle fatigue tests is equal to that of unidirectional tensile tests, the test frequency can be calculated using the following two equations:

(1) $$V=\frac{4L}{T}$$

(2) $$V=\dot{\varepsilon }\cdot L_{0}$$

where V is the operating speed of the testing machine beam, L is the variation in the gauge section of the specimen, $L_{0}$ is the extensometer gauge length, and $\dot{\varepsilon }$ is the strain rate. According to the calculation, when the strain amplitudes are 0.5%, 0.6%, 0.8%, and 1.0%, the LCF test frequencies are 0.125, 0.10417, 0.07813, and 0.0625, respectively. For each strain amplitude, at least three samples were performed. And the stress–strain hysteresis curve consists of 400 data points per cycle.

After the experiments, SEM (Nippon Electronics, Showa City, Tokyo, Japan) was used to detect the microstructure of the fatigue fracture surface. In addition, to obtain the grain information of the as-received material, an Electron Backscatter Diffraction (EBSD) test is conducted on the planned surface by FEI Quanta 450F SEM equipped with Oxford instruments HKL system (Thermo Fisher Scientific, Walther, MA, USA). Before the EBSD test, the test sample is first mechanically polished by SIC paper, followed by electrochemical etching. After that, the EBSD test is conducted with a step size of 1 μm at 15 kV.

In addition, to study the evolution behavior of dislocation structures before and after the fatigue test, the TEM test (FEI Talos F200X, Thermo Fisher Scientific, Walther, MA, USA) is used to observe the microstructure. Before the TEM test, a longitudinal foil is prepared near the fracture and then mechanically polished to a thickness of 100 μm. After being polished, the test samples are electrolytically thinned to a thickness of 20 μm. After that, the TEM test is conducted at 200 kV, and the Energy Dispersive Spectroscopy (EDS) system is used to measure the chemical compositions of the oxide inclusions. Moreover, the Bright-field/dark-field imaging modes are used to capture representative dislocation structures.

## 3. Results and Discussion

### 3.1. Initial Microstructure

**Figure 2:**
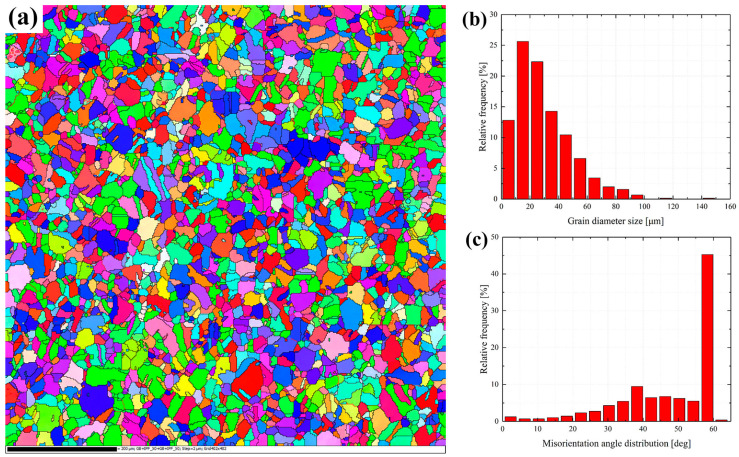
EBSD characterization of SS304: (**a**) inverse pole figures; (**b**) grain size distribution; (**c**) misorientation angle distribution.

**Figure 3:**
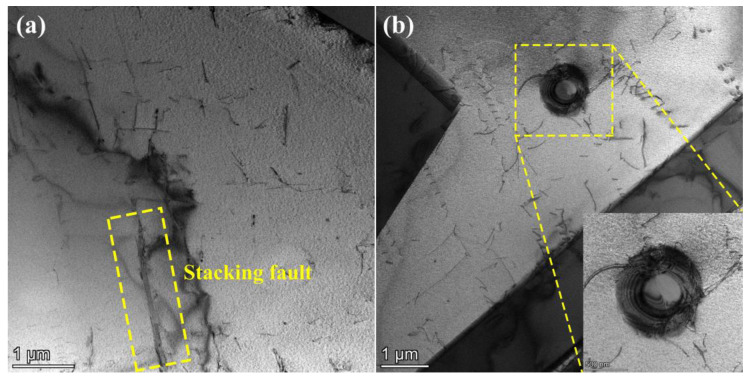
TEM characterization of SS304: (**a**) dislocations and stacking faults; (**b**) dislocations and precipitated phase, the yellow box indicates the precipitated phase.

In this study, to understand the crystallographic information of as-received material, the EBSD test is conducted. As presented in Figure 2, the inverse pole figure (IPF) of SS304 is shown, indicating that austenitic grains account for the majority. Meanwhile, it can be observed that there is little annealing twin of the as-received material in Figure 2a. Furthermore, the grain information including the grain size and misorientation angle distribution is also presented in Figure 2b,c. It can be observed that SS304 is overwhelmingly built by fine grains, with an average grain size of about 28.58 μm. Meanwhile, the histogram of the misorientation angle is concentrated on the right side of the x-coordinate axis, indicating that the high-angle grain boundaries are presented in the as-received material. Besides the EBSD information, the TEM microstructure characterization is also observed, as presented in Figure 3. As presented in Figure 3, dislocation lines are distributed haphazardly in the basic material, and stacking faults exist in the grains, like in previous studies [30,31]. Moreover, it can be observed that there is a tendency for dislocations to be emitted from the grain boundaries to the internal part, passing around the precipitated phrase, and having a tendency to move further. Additionally, the dislocation density is low within the as-received material.

### 3.2. Tensile Behavior under Various Temperatures

**Figure 4:**
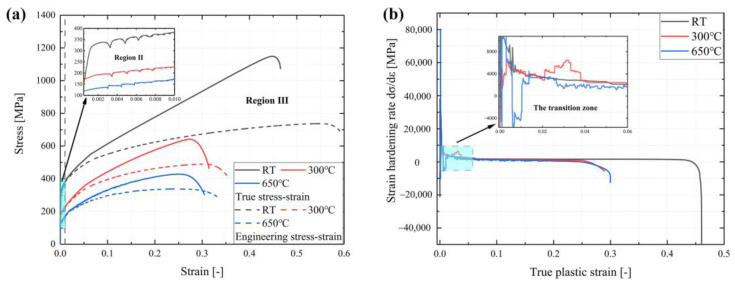
The tensile-related curves at RT, 300 °C, and 650 °C under 5 × 10^−3^ strain rate: (**a**) engineering and true stress–strain curves; (**b**) the strain-hardening rate curves.

The engineering and true stress–strain curves of SS304 at RT, 300 °C, and 650 °C are illustrated in Figure 4a. With the increase in temperature, the yield strength, tensile strength, and elongation decrease significantly. Tensile curves can be divided into three periods, i.e., an elastic region (linear elastic in Figure 4a, region Ⅰ), a micro-plastic (region Ⅱ), and a macro-plastic region (region Ⅲ). Differences in stress and strain are initially observed in the micro-plastic region, and the gaps in the macro-plastic region are rapidly widened, reflecting the decline in material properties at high temperatures. It is well known that austenitic stainless steel is affected by Portevin–Le Chatelier (PLC) instability at elevated temperatures [32], which is essentially the effect of dynamic strain aging (DSA). The macroscopic manifestation of this phenomenon is sawtooth flow stress [33], relating to accumulated plastic strain, as shown in Figure 4a region Ⅲ at 650 °C. Understood on a microstructure scale, the reason for PLC is the dynamic process of solute atoms blocking dislocation on barriers [34].

Moreover, as the strain grows, the flow stress increases, showing that SS304 has clear strain-hardening behavior at all temperatures. In Figure 4b, the Kocks–Mecking plot of the strain-hardening rate undergoes sharp fluctuations in the initial plastic transition zone before developing a stable strain-hardening rate during the work-hardening period. To assess the strain-hardening behavior, two evaluating approaches, the strain-hardening index and strain-hardening capacity, were chosen to describe its performance in tensile deformation. The former uses the Hollomon formula to calculate the parameters, and for the latter, the hardening capacity ($H_{c}$) can be considered as the ratio of tensile strength to yield strength. Two equations are as follows [35,36]:

(3) $$\sigma =\sigma_{Y}+k\varepsilon_{p}^{n}$$

(4) $$H_{c}=\frac{\sigma_{UTS}-\sigma_{Y}}{\sigma_{Y}}=\frac{\sigma_{UTS}}{\sigma_{Y}}-1$$

where k and n are strength coefficients and the strain-hardening exponent, respectively. Detailed tensile properties are displayed in Table 2 and each of the shown mechanical properties of SS304 drops with increasing temperature. The reason for this is that SS304, as a metal-stable austenitic alloy, undergoes martensitic transformation through atomic surface displacement under low temperatures, producing a positively correlated combination of high strength and ductility [37]. However, when the deformation temperature exceeds Md (the maximum temperature martensitic phase transformation occurs) at 322 K [38], the strain-induced martensitic phase transformation process is inhibited [39,40]. Below the Md temperature, plastic deformation causes parallel shear bands to appear in sub-stable austenitic, and with increasing strain, cross-shear bands develop where $\alpha^{\prime }$ martensite nucleation occurs [41]. Compared to RT, the tensile curves at 300 °C and 650 °C have weaker strength and ductility since the bcc-martensite is unfortunately lost as an effective barrier to dislocation movement in the FCC-austenite phase [42]. From 300 °C to 650 °C, the strength degrades but the ductility is similar depending on the micro-mechanism, as discussed in Section 3.4.

### 3.3. Cyclic Response and Hysteresis Loops

The cyclic response characteristic curve provides a visual representation of the change of the maximum cyclic stress amplitude with the number of cycles and the cyclic strengthening in LCF. Cyclic hardening and softening can be determined by examining the behavior in the stress amplitude with loading cycles in strain-controlled loading conditions. Some researchers have introduced the concept of the cyclic hardening or softening factor. For strain-controlled experiments, the hardening factor ($H_{e}$) [7,43] can be described as follows:

(5) $$H_{e}=\frac{\Delta \sigma_{s}-\Delta \sigma_{1}}{\Delta \sigma_{1}}$$

where $∆\sigma_{s}$ is the stress amplitude at the saturated cycle and $∆\sigma_{1}$ is the stress amplitude at the first cycle in the stable cycles. Clearly, $H_{e}>0$ demonstrates the cyclic hardening properties of the material, and $H_{e}<0$ is used to determine the cyclic softening.

**Figure 5:**
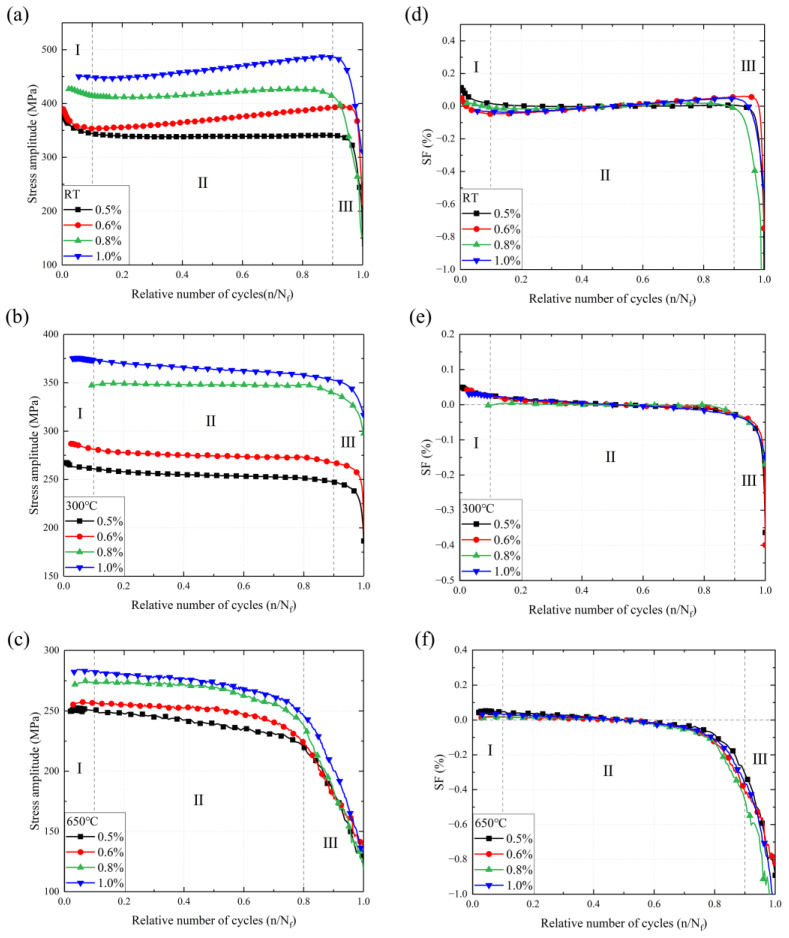
The stress amplitude and softening factor of SS304 at different temperatures: (**a**,**d**) at RT, (**b**,**e**) at 300 °C, (**c**,**f**) at 650 °C.

The cyclic stress response at RT, 300 °C, and 650 °C with different applied strain amplitudes reveals the cyclic mechanism of SS304 in Figure 5a–c, exhibiting two opposite conditions at RT and high temperatures. The stress amplitude of austenitic stainless steel can be divided into three classical stages, i.e., stage Ⅰ: a rapid decline zone due to the generation of plastic strain providing energy for crack initiation below 10% of total fatigue life; stage Ⅱ: the main stage of the cyclic stress response where the steady increase or decrease zone is on account of microcrack propagation; stage Ⅲ: a severe cyclic softening and failure zone after 90% of total fatigue life. Cyclic hardening behavior occurs in the RT stress amplitude plot, with a significant increase in stress amplitude and dynamic load carrying capacity with increasing cycles in Stage II. At 300 °C and 650 °C, however, there is continuous softening behavior throughout the fatigue process. The cyclic softening effect increases with the increasing strain amplitude at 300 °C and is particularly dramatic at 650 °C at arbitrary strain amplitudes. As the level of applied strain amplitude increases, the stress amplitude increases at all temperatures. On the contrary, the stress amplitude decreases with increasing temperature. At 300 °C, the large strain amplitude has a definite contribution to the cyclic softening of SS304. Temperature plays a dominant role in cyclic softening, especially at higher temperatures. In summary, the stress amplitude depends on the applied strain amplitude and temperature.

The drop in material properties attributable to temperature is critical, and the softening factor (SF) adopted to describe the cyclic hardening/softening behavior is as follows [44]:

(6) $$SF=\frac{\Delta \sigma -\Delta \sigma_{N_{f}/2}}{\Delta \sigma }$$

where ∆σ is the strain range ($\sigma_{max}-\sigma_{min}$), and $∆\sigma_{N_{f}/2}$ is the half-life stress range. As shown in Figure 5d–f, SF curves at all temperatures can be divided into three stages. Region Ⅰ and Ⅲ are cliff drop zones while Region Ⅱ collapses into curves approximating y = 0. The results suggest that SF is little affected by the strain amplitude but is really temperature sensitive.

**Figure 6:**
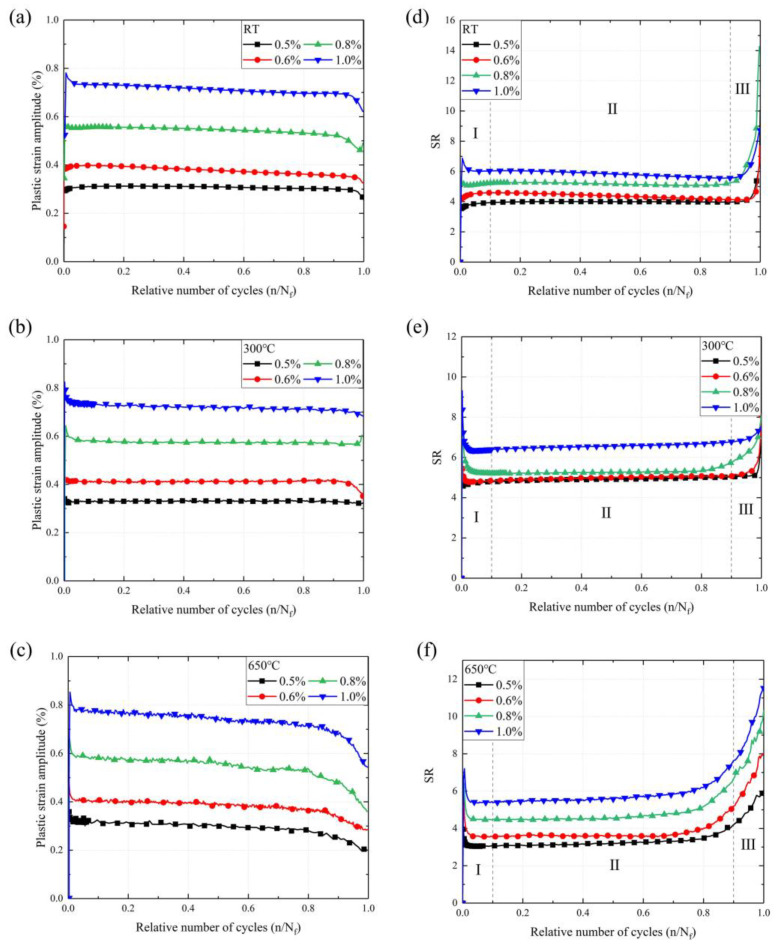
The plastic strain amplitude and strain ratio of SS304 at different temperatures: (**a**,**d**) at RT, (**b**,**e**) at 300 °C, (**c**,**f**) at 650 °C; Region I is the rapid descent zone, Region II is the Steady-state region, and Region III is the Rapid rise zone.

The plastic strain amplitude, which is correlated with the LCF life, is related to the applied strain amplitude at each temperature in Figure 6a–c. The plastic strain amplitude depends on the applied strain amplitude in LCF, with a higher total strain amplitude being followed by a higher plastic strain amplitude. In contrast, the effect of temperature on the plastic strain curves is mainly in the second half of the curves, where the proportion of stable plastic strain cycles to the total strain cycles decreases as the temperature increases. The softening behavior of SS304 caused by increasing temperature is responsible for the premature failure of the material. To further evaluate the differences in the plastic strain at three temperature modes, an empirical equation of the cyclic strain ratio (SR) is defined as follows [12,45]:

(7) $$SR=\frac{\Delta \varepsilon_{p}}{\Delta \varepsilon_{e}}=\frac{E\cdot \Delta \varepsilon }{\Delta \sigma }-1$$

where ∆σ is the total stress range, ∆ε is the total strain range, and *E* is the elastic module of SS304. Regardless of the situation in Figure 6d–f, the SR increases rapidly until the fatigue life reaches 10% in Region Ⅰ, and then failure occurs after the fatigue life approaches 90%. It can be observed that SR curves show a similar trend to the stress amplitude curves. The SR of the higher strain amplitude is greater than the lower strain amplitude at different temperatures. As the temperature increases, the differences between the SRs of different strain amplitudes become larger, indicating that the effect of temperature on the micro-plastic properties of the material is more powerful than that of the elastic properties.

**Figure 7:**
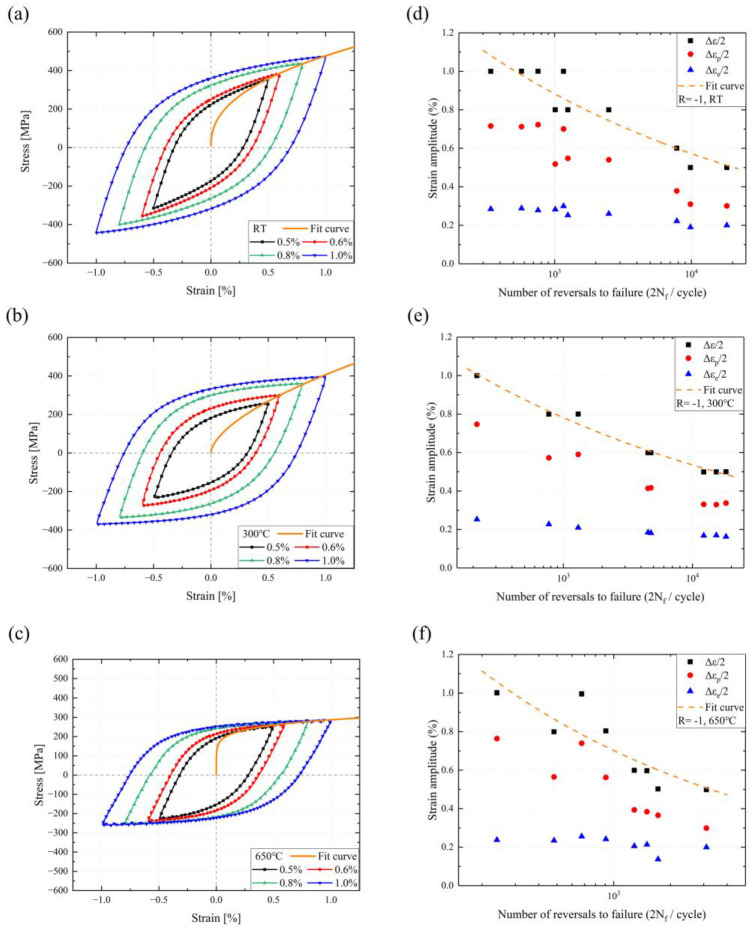
The 50% fatigue life hysteresis loops curves and strain fatigue life fitting curves of SS304 at different temperatures: (**a**,**d**) at RT, (**b**,**e**) at 300 °C, (**c**,**f**) at 650 °C.

The energy consumed during the fatigue life of SS304 is the plastic strain energy, expressed as the sum of the areas covered by the cyclic stress–strain hysteresis curves. The hysteresis loops at the 50% fatigue life at three temperatures are shown in Figure 7a–c. SS304 has well-rounded hysteresis curves with positive energy dissipation. As the temperature increases, the hysteresis curve changes from an elongated shuttle shape to a flat and fat one, owing to cyclic softening. Furthermore, to reflect the stress amplitude response under different strain amplitudes, the Ramberg–Osgood model is generally adopted to describe it, with the following equation [46,47]:

(8) $$\frac{\Delta \varepsilon }{2}=\frac{\Delta \varepsilon_{e}}{2}+\frac{\Delta \varepsilon_{p}}{2}=\frac{\Delta \sigma }{2E}+{\left(\frac{\Delta \sigma }{2K^{\prime }}\right)}^{\frac{1}{n^{\prime }}}$$

where $K^{\prime }$ and $n^{\prime }$ are the cyclic strain-hardening coefficient and exponent, respectively. The fitting parameters of the cyclic stress–strain curves at different temperatures are presented by the orange lines in Table 3. The fitting curves demonstrate that the softening effect of SS304 grows with increasing temperature. There is a tendency to transform from cyclic hardening to cyclic softening at 300 °C, as the parameters show a non-uniform change.

### 3.4. Microstructure Evolution at Various Temperatures

**Figure 8:**
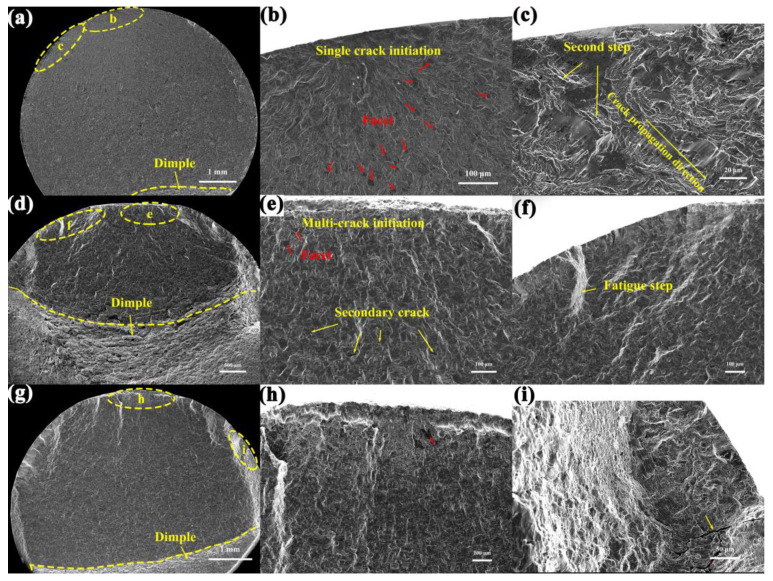
SEM images of surface damage features of SS304: (**a**–**c**) RT, Δε/2 = 0.6%; (**d**) RT, Δε/2 = 0.8%; (**e**–**g**) 300 °C, Δε/2 = 0.6%; (**h**,**i**) 650 °C, Δε/2 = 0.6%; the red and yellow arrows indicate the small facet and secondary crack, respectively.

Fatigue structure morphologies of SS304 are displayed in Figure 8, compared to the cyclic loading processes with the 0.6% controlled strain amplitude, consisting of the fatigue crack initiation, fatigue crack propagation, and momentary fracture zone. The area around the fatigue crack source zone is relatively flat, and the transition to the propagation zone river shapes and micro-porous patterns can be observed, with the transient zone showing the concentration of dimples representing plastic deformation. The fatigue crack initiation is always sited in or near the free surface, where two cases exist: a single initiation location at RT and multiple initiation locations at 300 °C and 650 °C in Figure 8b,e,h, respectively. This is the result of the degradation of material properties due to cyclic softening at high temperatures, where multiple fatigue crack initiations are not always in the same plane. In the mid-fatigue stage, the extension cracks intersect and join different planes through shear tearing, forming numerous secondary cracks and rough step shapes. In the earlier period of LCF, the rate of crack propagation is low, the opening and closing of the microcracks driven by tensile-compressive loading causes repeated friction around the crack initiations. Therefore, numerous small clear facets in the source area are most evident at RT, and the number of clear facets decreases dramatically at high temperatures due to microstructural degradation.

In summary, it can be derived that small clear facets and cyclic softening are factors influencing the initiation and expansion of LCF microcracks in SS304. The stress concentration caused by extrusion and the shear effect under the cyclic loading at RT leads to further localized plastic deformation damage, inducing microcrack initiation, creating clear facet morphology, and further directing stress concentration along facets. The effect of cyclic softening on LCF crack initiation is mirrored by the contiguous areas of crack initiation on the fracture surface as the temperature increases. This, coupled with the sharp reduction in the number of clear facets, suggests that the cyclic softening characteristics are gradually replacing small planar morphology as the main factor in the low circumferential fatigue fracture of SS304, manifested by microstructural degradation at elevated temperatures.

**Figure 9:**
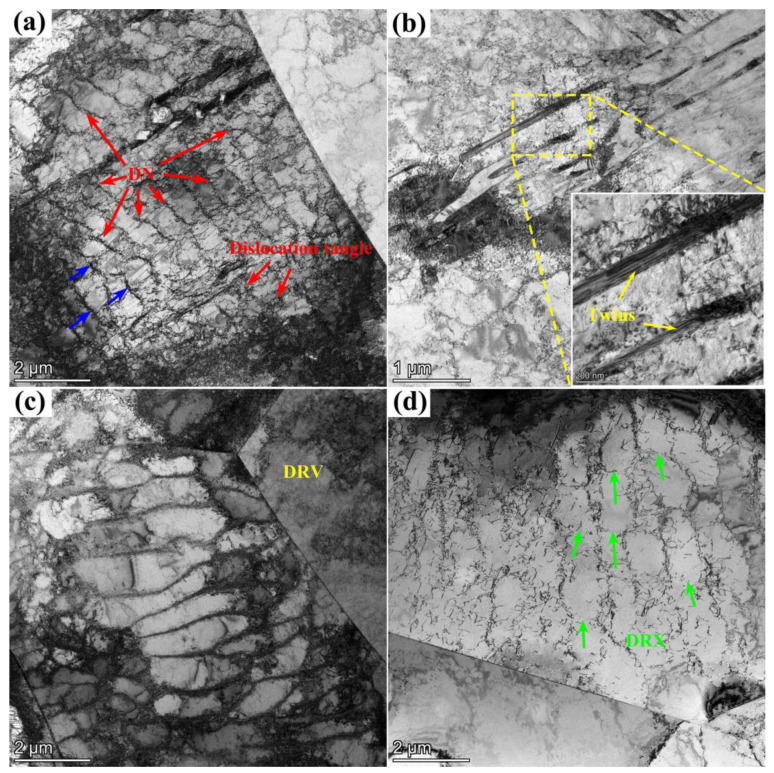
TEM micrographs of SS304 under 0.6% strain amplitude at different temperatures: (**a**,**b**) RT; The blue arrows and the yellow box indicate the dislocation wall and twins; (**c**) 300 °C; (**d**) 650 °C.

Additionally, to further understand the microstructure evolution during LCF, the effect of temperature on SS304 is presented in Figure 9. TEM micrographs were selected at the locations of the fatigue crack initiation under 0.6% strain amplitude at RT, 300 °C, and 650 °C. It is well-known that dislocation theory is the basis of microscopic plastic deformation and explains the macroscopic behavior of materials under different loading conditions through dislocation movements and other lattice defects. High-density dislocation along and within the matrix grain boundary can be observed at all temperatures. For metallic materials, dislocation strengthening is one of the most fundamental strengthening strategies, and the storage and annihilation of dislocation in plastic deformation affects the macroscopic work-hardening and dynamic softening effects of the material. In the Bailey–Hirsch relationship [48], the dislocation density is proportional to dislocation strengthening.

Figure 9a indicates that high-density dislocation forms a typical dislocation network (DN), which impedes the movement of dislocation and grain boundaries. The faint approximate cellular structure also appears in the upper right corner of the image. Meanwhile, dislocation tangles are observed around the carbonaceous precipitates (as the blue arrows show). The precipitates act as local pegs for the free dislocations, which are often followed by the nucleation prismatic loops or dipoles intensifying the hardening characteristic of the material [49]. Apart from this, a large amount of planar slip also exists. This is because SS304 has a low stacking fault energy, where dislocations are somewhat confined to its slip planar, and the interaction of slip systems leads to additional cyclic hardening [50]. Deformation twins mostly occur in regions of homogeneous distortion in polycrystals, and the deformation requires greater shear stress than slip. The critical shear stress required for deformation twinning is more easily reached during super cyclic hardening, providing the impetus for twin nucleation, and driving the formation of deformation twins. Deformation twins can be identified at RT in Figure 9b. The presence of deformation twins has the pining effect and reduces the average distance of free dislocations, providing strong dislocation storage capacity and impeding dislocation movement [51]. A similar phenomenon concerning deformation twins has been discussed in previous articles [52,53]. Given these mechanisms, it is possible to improve the plastic deformation resistance and maintain the stability of the cyclic hardening in LCF at RT.

As the temperature increases, the dislocation substructure changes until the dislocation cells form, which shows the balance between dislocation multiplication and annihilation activity. The dense dislocation cells reflecting the cyclic saturation state can be observed in Figure 9c. During cyclic deformation, the tangled dislocations are supplemented by separated atoms to form cellular substructures that act as soft barriers for dislocation motion during cyclic deformation [30], which leads to significant back stress and a planar deformation mechanism [31]. The presence of dislocation cells, commonly associated with the final stage of recovery, occurs later in the cyclic softening period of FCC alloys [54]. Although dislocation cellular structures are seen at both RT and 300 °C, there is a specific gravity relationship between grains that have already been in the dynamic recovery process and cellular development stages, while others remain in the hardening stage because of the grain heterogeneity in microstructure evolution [55]. If the former proportion is substantially ahead, the LCF of SS304 is characterized by cyclic hardening, and if the latter is dominant, cyclic softening is visible on the macroscopic scale. In summary, the rearrangement of dislocations to a low-energy state organization during dynamic recovery, the formation of sub-grains, and the reduction in dislocation density are responsible for the slight cyclic softening of SS304 at 300 °C.

Furthermore, under plastic deformation conditions, the cellular structures suffer from non-negligible hetero-deformation-induced stress, which inhibits dislocation motion and plays a role in promising fatigue properties [56]. When the temperature reaches 650 °C, dynamic recrystallization (as the green arrows show) replaces dynamic recovery as the primary mode of dynamic softening. In Figure 9d, it is provided that the number of in-place dislocation pileups has a greater drop than that at 300 °C. The rate of dislocation annihilation relative to dislocation annihilation increases and the low-angle grain boundaries (LAGBs) gradually disappear. The absence of cell boundaries can be explained by solute homogenization and dislocation migration, diminishing the twinning stress required to trigger twinning, so that a similar number of deformation twins as at RT are not expected at high temperatures [57].

Taken together, SS304 exhibits cyclic hardening at RT, where dislocation migration is influenced by DN, precipitation phases, and deformation twins, and shows cyclic softening at high temperatures, where the relative rate of dislocation annihilation rises gradually, influenced by dynamic recovery and recrystallization. The higher the temperature, the more marked the degree of thermal softening. The effect of temperature on SS304 is mainly manifested in the way dislocations evolve, causing microscopic mechanism changes and opposed cyclic properties at room and high temperatures.

### 3.5. Fatigue Life Evaluation

It is observed that a linear relationship exists between the logarithm of the fatigue life ($N_{f}$) and the strain amplitude ($\sigma_{a}$). To accurately predict the LCF life of SS304, the Basquin and Manson–Coffin model is chosen based on the experimental data, as follows [58,59,60]:

(9) $$\frac{\Delta \varepsilon }{2}=\frac{\Delta \varepsilon_{e}}{2}+\frac{\Delta \varepsilon_{p}}{2}=\frac{\sigma_{f}{}^{\prime }}{E}{\left(2N_{f}\right)}^{b}+\varepsilon_{f}{}^{\prime }{\left(2N_{f}\right)}^{c}$$

where $\varepsilon_{f}\prime$ and b present the fatigue strength coefficient and exponent, $\varepsilon_{f}\prime$ and c denote the fatigue ductile coefficient and exponent, and ${2N}_{f}$ expresses the number of reversals to failure. The elastic strain component dominates the LCF behavior at low strain amplitudes, while the plastic strain component is the major fatigue driving force at low cyclic life. The half-strain life under the strain-controlled condition at different temperatures is plotted in Figure 7, and the relevant parameters are listed in Table 4. The fitting parameters at different temperatures are provided in Table 4, and the semi-logarithmic curves of strain amplitude and the number of reversals to failure can be found in Figure 7d–f. The elastic strain at different temperatures and strain amplitudes do not differ significantly and are stable between 0.2% and 0.3%. Compared to the elastic strain amplitude, the plastic strain amplitude is more discrete and dominant in the total strain amplitude. This indicates that the fatigue life of SS304 is influenced by the material strength but depends on the material plasticity.

**Figure 10:**
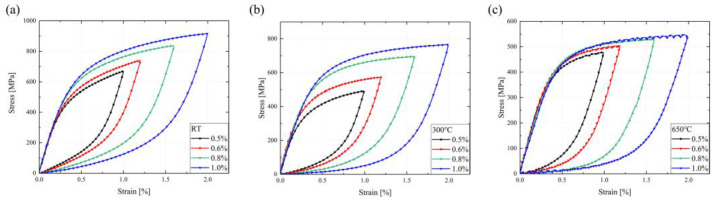
Convert the hysteresis loops to the axis origin at different temperatures: (**a**) RT, (**b**) 300 °C, (**c**) 650 °C.

The crack initiation and propagation period are primarily the process of energy absorption and consumption. Since fatigue damage is generally caused by plastic cyclic strain, it is important to study the dissipated plastic strain energy by energy methods. The energy methods are employed as the focus for the research of fatigue life prediction methods, considering the effects of applied stress and strain on the fatigue life simultaneously. In Figure 10, the axis origin is converted to the tip of the negative (compressive) cycle of the hysteresis loop. The difference in the form of the hysteresis loops depends on the applied cyclic strain amplitude. The area of the per hysteresis loop represents the energy dissipated in a single cycle, which is converted into heat energy dissipated through local damage (e.g., plastic deformation, cracking, etc.). With the increase in the applied strain amplitude, the fatigue stress and strain obviously grow, and the plastic energy consumed by SS304 increases per cycle. However, as the temperature increases, the sensitivity of the material to the strain amplitude decreases. At the same strain amplitude, the ability of the material to dissipate energy decreases with increasing temperature, which is not conducive to maintaining structural and property stability under fatigue loading. Furthermore, SS304 cannot follow the mashing-type description [23], because the trajectory of the upward hysteresis curves coincides with each other.

**Figure 11:**
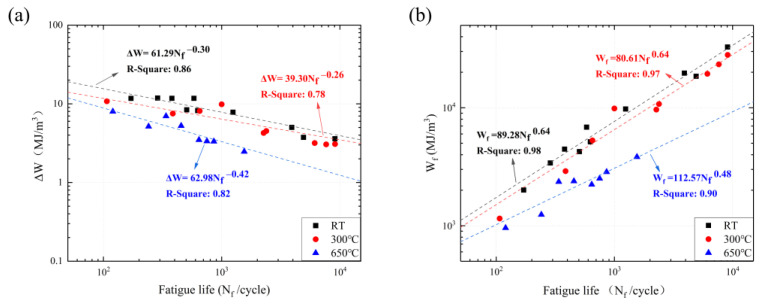
Plastic strain energy per cycle (**a**) and cumulative plastic strain energy (**b**) of SS304 at RT, 300 °C, and 650 °C.

According to the cyclic cumulative strain energy model for non-mashing material, the plastic strain energy per cycle and the cumulative plastic strain energy at failure are as follows [22,23,61,62]:

(10) $$\Delta \varepsilon_{p}={\left(\frac{\Delta \sigma }{2K^{*}}\right)}^{\frac{1}{n^{*}}}$$

(11) $$\Delta W=\frac{1-n^{*}}{1+n^{*}}\Delta \sigma \cdot \Delta \varepsilon_{p}+\frac{2n^{*}}{1+n^{*}}\delta \sigma_{0}\cdot \Delta \varepsilon_{p}$$

(12) $$\delta \sigma_{0}=\Delta \sigma -2K^{*}{\left(\frac{\Delta \varepsilon_{p}}{2}\right)}^{n^{*}}$$

(13) $$W_{f}=\Delta W\cdot N_{f}$$

where ∆W and $W_{f}$ are the plastic strain energy per cycle and cumulated plastic strain energy, respectively; $K^{*}$ and K, $n^{*}$ and $n^{\prime }$ are different while the fitting values are similar, $\delta \sigma_{0}$ is a quantity equal to the increase in the proportional stress range caused by the non-mashing loop. The calculated results and associated fitting curves are shown in Figure 11. The plastic strain energy per cycle and the cumulative plastic strain energy decreases with increasing temperature. Whether SS304 exhibits cyclic hardening behavior at RT or cyclic softening behavior at HT, the cyclic plastic strain energy is monotonically reduced with an increase, which is attributed to the accumulation of material damage and crystal defects caused by the cyclic loading process. With the increasing number of cycles, dislocation density at the grain boundary grows in the SS304 crystals, intensifying the resistance to dislocation movement. At the same time, the growing dislocation density will produce stress concentration at grain boundaries. When the stress value exceeds a particular value, fatigue microcracks appear and expand to macrocracks leading to fracture and eventually failure. The development and expansion of microcracks is the source of the reduction in cyclic plasticity of SS304. These are the general microscopic reasons for the decreasing fatigue cycles; a more detailed analysis is given in Section 3.4.

To assess the effect of temperature on fatigue life at R = −1, the following damage energy model is explored by defining the proportion of damage in a single cycle. Through a life prediction with hysteresis energy, the damage parameter D can be obtained as follows [63]:

(14) $$D_{i}={\left(\frac{W_{i}}{W_{0}}\right)}^{\beta }$$

(15) $$\sum_{i=1}^{N_{f}}D_{i}=1$$

of which $D_{i}$ and $W_{i}$ represent the damage parameter and hysteresis energy of the ith cycle; $W_{0}$ is a common material constant of this model. To simplify the hysteresis energy model, it is practical to replace $W_{i}$ with $W_{s}$, a constant value of the saturation hysteresis energy. The improved model can be illustrated in the equation below.

(16) $$W_{s}=W_{0}\cdot N_{f}{}^{-\frac{1}{\beta }}$$

**Figure 12:**
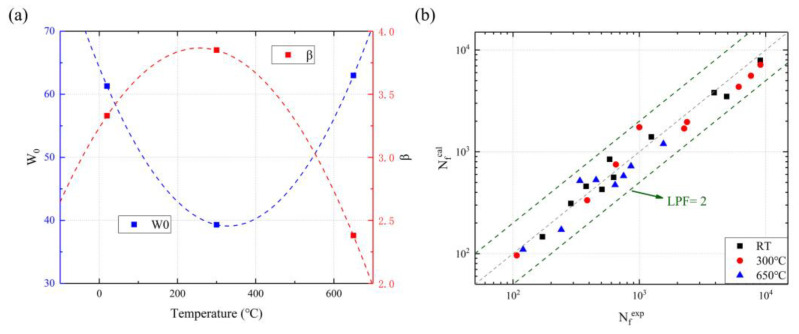
The parameters (**a**) and the life prediction (**b**) of the SS304 modified model.

Where both $W_{0}$ and β can be expressed as a fitting quadratic polynomial with the temperature in Figure 12.

(17) $$W_{0}=2.32052\times 10^{-4}T^{2}-0.15279T+64.25303$$

(18) $$\beta =-9.61451\times 10^{-6}T^{2}+0.00493T+3.23517$$

By combining the three equations above, the modified the LCF life prediction is available as follows:

(19) $$W_{f}=\left(2.32052\times 10^{-4}T^{2}-0.15279T+64.25303\right)\cdot N_{f}{}^{\frac{1}{9.61451\times 10^{-6}-0.00493T-3.23517}+1}$$

of which $W_{0}$ and β exhibit an opposite changing path. In principle, $W_{0}$ is defined as the fatigue toughness of SS304, and the larger $W_{0}$ is, the greater its resistance to fatigue damage. However, the other important parameter β, called the damage transition index, is the reduction and dispersion of fatigue damage. The different material properties observed at 300 °C are worthy of further study under three temperatures.

To assess the rationality and professionalism of the modified LCF model, the life prediction factor (IPF) is proposed to conduct an error analysis,

(20) $$LPF=MAX\{\frac{N_{f}^{\exp}}{N_{f}^{cal}},\frac{N_{f}^{cal}}{N_{f}^{\exp}}\}$$

where $N_{f}^{exp}$ and $N_{f}^{cal}$ are the experimental and calculative data of the fatigue life. The result demonstrates that the modified model accurately predicts the LCF life of SS304 at RT, 300 °C, and 650 °C.

## 4. Conclusions

In this study, to understand the cyclic response behavior, fatigue failure behavior, and microstructural evolution of SS304 during cyclic loading, systematic analyses including uniaxial tensile tests and low-cycle fatigue tests are conducted at RT, 300 °C, and 650 °C. Several conclusions can be obtained as follows:

(1) The tensile properties of SS304 decrease with increasing temperature and show work-hardening characteristics at all temperatures. However, the stress amplitude of SS304 presents cyclic hardening in RT and cyclic softening at 300 °C and 650 °C. The softening factor is minimally affected by the strain amplitude but is temperature sensitive.
(2) The fatigue crack initiation mechanism of SS304 is single-point and multi-point initiation at RT and high temperatures, respectively. Fatigue crack initiation is controlled by a clear facet at RT and cyclic softening at 300 °C and 650 °C. Therefore, as the temperature increases, cyclic softening gradually replaces the clear facet as the primary mechanism for crack initiation.
(3) Cyclic hardening of SS304 at RT is related to the forming dislocation nets, pinning dislocation by carbon-containing precipitates, low stacking fault energy, and deformation twins. In addition, dynamic recovery and dynamic recrystallization are proposed to explain the cyclic softening behavior at 300 °C and 650 °C. Dislocation morphology transforms from dislocation tangles and walls at RT to dislocation cells at 300 °C to dislocation annihilation, revealing that the effect of temperature on cyclic properties of SS304 originates from dislocation evolution.
(4) After determining the non-mashing type of SS304, the quadratic relationship between the model parameters ($W_{0}$ and β) and temperature is constructed based on the hysteresis energy model. The LPF analysis predicts that the experimental data possess accuracy and validity, which benefits the anti-fatigue design.

## Figures and Tables

**Table 1:** Chemical composition of SS304 (wt.%).

C	Si	Mn	P	S	Cr	Ni	Fe
0.010	0.52	1.03	0.020	0.010	18.34	8.44	balance

**Table 2:** Tensile properties of SS304 at RT, 300 °C, and 650 °C.

Temperature (°C)	Elastic Modulus (GPa)	Yield Strength (MPa)	Tensile Strength (MPa)	*n*	Hardening Capacity
RT	270.79	319.82	1149.01	0.32	2.59
300 °C	239.00	181.20	641.53	0.30	2.54
650 °C	153.17	125.03	435.64	0.26	2.48

**Table 3:** Cyclic stress–strain properties of SS304 at RT, 300 °C, and 650 °C.

Temperature (°C)	$K^{\prime }$ (MPa)	$n^{\prime }$	$n^{*}$	$K^{*}$	R^2^
RT	476.46	0.41	0.35	522.84	0.98
300	406.56	0.60	0.54	453.05	0.97
650	286.96	0.15	0.17	299.52	0.88

**Table 4:** Fitting parameters of the Basquin and Manson–Coffin model at RT, 300 °C, and 650 °C.

Temperature (°C)	$\sigma_{f}^{\prime }$ (MPa)	$\varepsilon_{f}^{\prime }$	b	c	$R^{2}$
RT	768.36	3.25	−0.09	−0.19	0.87
300	660.17	2.43	−0.11	−0.16	0.98
650	424.68	5.09	−0.08	−0.29	0.76

## Data Availability

The data that support the findings of this study are available from the corresponding author upon reasonable request.
